# Inquiries Concerning Several Chemical Preparations Used in Physic; Read at the Public Meeting of the Faculty of Physic at Paris, Nov. 5, 1778

**Published:** 1781-11

**Authors:** 


IV. *Recherches sur quelques preparations chemiques, appliquées a l'usage de la medecine; lües a la seance publique de la faculté de medecine de Paris le 5 Novembre* 1778; *par* M. Majault, *D. R. de la dite faculté; augmentées depuis de plusieurs observations en refutation de ce qu'on a publié sur les propriétés de l'alkali volatil fluor, celles du savon et du foyer de soufre.* i. e. *Inquiries concerning several chemical preparations used in physic; read at the public meeting of the faculty of physic at Paris, Nov.* 5, 1778, *by* M. Majault, *Doctor Regent of the said faculty; to which are now added, several remarks in refutation of what has been published relative to the properties of volatile alkali fluor, and those of soap and liver of sulphur.* 8vo. Paris, 1779. 50 pages.

THIS experienced writer, who has for many years officiated as physician to the Hotel Dieu, is very severe in his strictures on certain chemical preparations that have lately been introduced into the practice of physic. He confines himself chiefly to the volatile alkali fluor, which has been so warmly recommended by M. Sage, in apoplexy, asphyxy, &c. to the use of soap as an antidote to aqua fortis; and to the hepar sulphuris as an antidote to arsenic, and ſome other mineral poiſons. With regard to the firſt of theſe, our author begins with cautioning the practitioner not to confide in it in the treatment of apoplexy. He obſerves, that if the diſeaſe is owing to plethora, by adminiſtering the volatile alkali we only ſacrifice valuable moments, which might be better employed in bleeding and in other evacuations, the effects of which would be more beneficial than ſuch a ſtimulant, in a diſeaſe in which every thing that accelerates the motion of the blood may prove hurtful; that on the other hand, if the apoplexy is of the ſerous kind, by repoſing wholly on this irritating vapour we may be liable to omit other means of relief, ſuch as vomits, purgatives, and the ſtimulus of bliſters, the long continued irritation of which, he contends, renders them far preferable to that of the volatile fluor, the effects of which are only momentary: hence, concludes M. Majault, in the humoral apoplexy the alkali fluor leaves the cauſe of the diſeaſe, and of courſe the diſeaſe itſelf, in its full force; and in the ſanguineous apoplexy the ſame remedy increaſes both the cauſe and the effects of the complaint. M. Sage has recommended his remedy in aſphyxy, a diſeaſe which he pretends is owing to a mephitic phitic acid acting on the glottis and lungs, and putting a ſtop to their functions. "Neutralize "this acid—ſays M. Sage—and you recover the "patient; and how can ſuch a neutralization "be effected—adds he—more ſpeedily, or with "more certainty, than by means of the volatile "alkali?"—In anſwer to this curious reaſoning the writer of the work before us obſerves, that in order to produce ſuch an effect, it is at leaſt neceſſary that the alkali ſhould unite with the acid. Now—ſays he—in a ſtate of aſphyxy, the patient either does or does not breathe.—If he does breathe, the atmoſpheric air, by paſſing into the lungs will ſoon remove that which is mephitic, and in ſuch caſe the ſpecific will be of no uſe. If on the other hand the patient does not breathe, how will it be poſſible to introduce air impregnated with the volatile alkali fluor into the lungs? and yet it is evident there can be no neutralization unleſs the alkali and acid are united. Of courſe—adds our author—this pretended remedy can be of no uſe, though in ſome caſes it may prove dangerous; as, for inſtance, may happen, if at the inſtant the patient begins to breathe the atmoſpheric air is ſufficiently impregnated with the volatile alkali to neutralize the mephitic acid contained in the lungs; becauſe caufe by fuch an application the patient will probably be fuffocated.

After having confidered the volatile alkali, our author proceeds to another remedy announced in the *Journal de Medecine* for May 1778. This remedy, which confifts of a folution of foap, has been recommended as a fpecific to counteract the baneful effects of aqua fortis taken internally. If this folution is mixed with aqua fortis, a neutral falt will no doubt be formed by a combination of the nitrous acid with the alkali that enters into the compofition of the foap, and the former will of courfe be inactive. But, fays M. Majault, the nitrous acid cannot pafs with impunity through the mouth and œfophagus, or remain inactive in the ftomach; it corrodes, it inflames all the parts it touches, and it is certain that a folution of foap cannot obviate or remedy thefe effects. If, when taken into the ftomach it neutralizes fome portion of the acid, ftill it will irritate the inflamed membranes and increafe the pain; thefe truths, our author obferves, are every day proved by fimple practical facts, fo as to admit of no doubt. From thefe facts, he adds, it is evident, that promifes of a neutralization which fhall prevent or remove the effects of cauftics, are nugatory, and that mucilaginous laginous remedies in different forms, and amongſt others a lohoch compoſed of the yolk of an egg, gum arabic, and a large proportion of abſorbent earth, are the beſt adapted to theſe melancholy caſes.

Our author next proceeds to the refutation of certain doctrines advanced by M. Touſſaint, in his work entitled “ *Contrepoiſons de l'arſenic, &c.*” According to that writer all the hepars are antidotes to theſe mineral poiſons. M. Majault has particularly confined himſelf to what M. Touſſaint has ſaid of the effect of the hepar ſulphuris on arſenic. The reſult of this combination, ſays the latter, is a kind of orpiment ſo overloaded with ſulphur, and rendered ſo mild by the intimate manner in which the arſenic is united with it, that it becomes incapable of doing harm. An accurate deſcription of orpiment, and a recital of its dreadful effects when any one has had the misfortune to ſwallow it, are the arguments employed by our author to deſtroy this erroneous and dangerous aſſertion. He adds, that in theſe alarming caſes he has experienced the beſt effects from the mucilaginous lohoch abovementioned, combined with twenty drops of the eſſential oil of aniſeed. This remedy, which has long been ſucceſsfully adminiſtered at the Hotel

Dieu

Dieu to patients poiſoned with arſenic, was firſt preſcribed, it ſeems, in that hoſpital by the late M. Payen.

